# Identification of risk groups for mental disorders, headache and oral behaviors in adults during the COVID-19 pandemic

**DOI:** 10.1038/s41598-021-90566-z

**Published:** 2021-05-26

**Authors:** Mieszko Wieckiewicz, Dariusz Danel, Maciej Pondel, Joanna Smardz, Helena Martynowicz, Tomasz Wieczorek, Grzegorz Mazur, Robert Pudlo, Gniewko Wieckiewicz

**Affiliations:** 1 Department of Experimental Dentistry, Wroclaw Medical University, 26 Krakowska St., 50-425 Wroclaw, Poland; 2 Department of Anthropology, Hirszfeld Institute of Immunology and Experimental Therapy, Polish Academy of Sciences, 12 Rudolfa Weigla St., 53-114 Wroclaw, Poland; 3 Department of Business Intelligence in Management, Wroclaw University of Economics and Business, 118-120 Komandorska St., 53-345 Wroclaw, Poland; 4 Department of Internal Medicine, Occupational Diseases, Hypertension and Clinical Oncology, Wroclaw Medical University, 213 Borowska St., 50-556 Wroclaw, Poland; 5 Department and Clinic of Psychiatry, Wroclaw Medical University, 10 Pasteura St., 50-367 Wroclaw, Poland; 6 Chair and Clinical Department of Psychiatry in Tarnowskie Gory, Medical University of Silesia, 49 Pyskowicka St., 42-612 Tarnowskie Gory, Poland

**Keywords:** Health care, Risk factors

## Abstract

The dramatically changing situation during COVID-19 pandemic, is anticipated to provoke psycho-emotional disturbances and somatization arising from the current epidemiological situation that will become a significant problem for global and regional healthcare systems. The aim of this study was to identify the predictors, risk factors and factors associated with mental disorders, headache and potentially stress-modulated parafunctional oral behaviors among the adult residents of North America and Europe as indirect health effects of the COVID-19 pandemic. This may help limit the long-term effects of this and future global pandemic crises. The data were collected from 1642 respondents using an online survey. The results demonstrated increased levels of anxiety, depression, headache and parafunctional oral behaviors during the COVID-19 pandemic in both North American and European residents. The results of this study facilitated the definition of the group most predicted to experience the aforementioned secondary effects of the pandemic. This group included females younger than 28.5 years old, especially those who were single, less well educated and living in Europe. In case of this and other global crises this will allow faster defining the most vulnerable groups and providing rapid and more targeted intervention.

## Introduction

Coronavirus disease 2019 (COVID-19) is a severe respiratory syndrome caused by the new betacoronavirus severe acute respiratory syndrome coronavirus 2 (SARS-CoV-2)^1–3^. The typical clinical manifestations of COVID-19 are nonspecific and can mimic other diseases resembling flu symptoms^2,4–7^. Disease onset may result in progressive respiratory failure with alveolar damage and, in some cases, death^2^. Severe disease is more likely in elderly people, people with impaired immunity and those suffering from respiratory system diseases, cardiovascular diseases, cancer and diabetes^8^.

Indisputably, COVID-19 has already had a tremendous impact on regional and global health systems and economies. In many countries, bans and restrictions have been introduced to stop the spread of the virus^6^. However, the direct threats to physical health and life posed by COVID-19 are not the only impacts of the pandemic on worldwide health care systems. Certain aspects of the pandemic are believed to have major impacts on the mental health of the population. The need for isolation and the related economic issues and the fear of the possibility of the infection and death of immediate family members are undoubtedly significant in the context of the mental health of the global population^9–12^. Moreover, due to the dramatically changing situation, it is anticipated that psycho-emotional disturbances, somatization (which can manifest as a headache) and parafunctional oral behaviors (which can contribute to orofacial pain and temporomandibular disorders) arising from and intensified by the current epidemiological situation will become significant problems for global and regional healthcare systems in the future^9,11,12^. The available research show that socio-demographic and socio-economic factors such can be associated with adverse effects of the pandemic^13–15^. The lockdown situation unfortunately hampers the performance of extensive and sound research. Conducting online surveys seems to be the only safe large-scale solution available. Some evidence of COVID-19-related mental health issues and headache in the general public has been published^10–27^. Most of these studies were surveys showing increased symptoms of depression, anxiety, and stress related to COVID-19, mainly as a result of psychosocial stressors such as fear of negative health impacts, the loss of life and economic issues. The results of the abovementioned studies have been heterogeneous, probably because of differences in methods, locations and timing with regard to the stage of the pandemic. There have been only a few studies published regarding the influence of the pandemic on oral behaviors, such as bruxism^28,29^. None of the results of the published surveys investigated headache as a symptom of somatization related to the COVID-19 pandemic.

All the aspects of the COVID-19 pandemic discussed above and the insufficiency of well-documented and reliable research on its global impacts on mental health and headache indicate the need for a large-scale study on the indirect effects of the chronic fear induced by the pandemic. In the present study, we aimed to identify the predictors, risk factors and factors associated with mental disorders, headache and potentially stress-modulated parafunctional oral behaviors among the adult residents of North America and Europe using validated questionnaires administered as an online survey during the COVID-19 pandemic. We used author sociodemographic survey, Hospital Anxiety and Depression Scale (HADS), Migraine Disability Assessment Questionnaire (MIDAS) and Oral Behavior Checklist (OBC). Furthermore, we performed a decision tree analysis to identify multidimensional dependencies among the investigated characteristics. We hope that this study will help prepare global and local healthcare systems by enabling the identification of high-risk groups and therefore the more effective prevention of the secondary effects of the pandemic.

## Results

### Background characteristics of the sample

During the study period, a total of 1642 subjects responded to the questionnaire. In total, 1130 were from North America and Europe, and 99.91% (N = 1129) fully completed the questionnaire; 843 subjects from North America responded to the questionnaire, out of whom 100% (N = 843) fully completed it, and 287 subjects from Europe responded to the questionnaire, out of whom 99.65% (N = 286) fully completed it. The groups of respondents (North America and Europe) were adults; 47.74% (N = 539) were men and 52.26% (N = 590) were women. Among the respondents from North America, 43.42% (N = 366) were men and 56.58% (N = 477) were women. Among the respondents from Europe, 60.49% (N = 173) were men and 39.51% (N = 113) were women. The age of the respondents ranged from 18–72 years old, with a mean age ± standard deviation (SD) of 32.59 ± 9.18 years. The age of respondents from North America ranged from 18 to 72 years old, with a mean age ± SD of 32.65 ± 9.11 years. The age of respondents from Europe ranged from 18 to 66 years old, with a mean age ± SD of 32.09 ± 9.37 years.

#### Participant age

We observed statistically significant negative correlations between participant age and the MIDAS score (r(1129) = −0.08, *p* < 0.007), HADS-Anxiety (HADS-A) score (r(1129) =−0.14, *p* < 0.0001), HADS total score (r(1129) = −0.09, *p* = 0.002) and OBC score (r(1129) = −0.24, *p* < 0.0001). Older participants experienced less disability from migraines and less anxiety, obtained lower scores on the general HADS scale and reported a lower intensity of oral behaviors. The association between respondent age and HADS-Depression (HADS-D) scores was statistically nonsignificant (r(1129) = -0.03, *p* = 0.40).

#### Participant gender

We found statistically significant differences between the genders with respect to the MIDAS score (F_Welch_(1, 1055.99) = 26.45, *p* < 0.00001), HADS-D score (F(1, 1127) = 10.57, *p* = 0.001), HADS-A score (F_Welch_ (1, 1086.59) = 63.81), *p* < 0.0001), HADS total score (F_Welch_ (1, 1073.79) = 39.66, *p* < 0.0001) and OBC score (F(1, 1127) = 29.55, *p* < 0.0001). In all cases, women had higher scores than men. Detailed results are presented in Table 1.

**Table 1:** Mean scores for the MIDAS, HADS-D, HADS-A, HADS total and OBC in men and women (SD – standard deviation).

	MIDAS		HADS-D		HADS-A		HADS total		OBC	
	Mean	SD	Mean	SD	Mean	SD	Mean	SD	Mean	SD
Men (n = 539)	14.13	33.30	7.64	4.29	10.66	4.68	18.30	8.27	21.51	10.39
Women (n = 590)	26.64	47.71	8.44	3.95	12.79	4.22	21.23	7.24	24.82	10.06
Sig	*p* < .00001		*p* = .001		*p* < .0001		*p* < .0001		*p* < .0001	
Total (n = 1129)	20.67	41.91	8.06	4.13	11.77	4.57	19.83	7.88	23.24	10.35

SD—standard deviation, MIDAS—Migraine Disability Assessment, HADS—Hospital Anxiety and Depression Scale, OBC—Oral Behaviors Checklist, sig.—*p*-value for the statistical significance of the analyzed differences (see main text for details).

#### Europe vs. North America

Whereas European and North America respondents had similar HADS-D scores (F_Welch_ (1, 459.0) = 1.39, *p* = 0.24), there were statistically significant differences between European and North American respondents in the MIDAS score (F_Welch_(1, 884.47) = 20.94, *p* < 0.00001), HADS-A score (F(1, 467.32) = 66.68, *p* < 0.0001), HADS total score (F_Welch_ (1458.49) = 27.32, *p* < 0.0001) and OBC score for intensity (F(1, 1127) = 28.35, *p* < 0.0001). In all cases, North American residents scored higher than Europeans (Table 2).

**Table 2:** Mean scores for the MIDAS, HADS-D, HADS-A, HADS total and OBC in European and North American residents (SD—standard deviation).

	MIDAS		HADS-D		HADS-A		HADS total		OBC	
	Mean	SD	Mean	SD	Mean	SD	Mean	SD	Mean	SD
Europe (n = 286)	13.21	25.42	7.80	4.39	9.86	4.64	17.66	8.29	20.45	9.83
North America (n = 843)	23.19	45.92	8.14	4.04	12.42	4.37	20.56	7.60	24.18	10.36
Sig	*p* < .00001		*p* = .24		*p* < .0001		*p* < .0001		*p* < .0001	
Total (n = 1129)	20.67	41.91	8.06	4.13	11.77	4.57	19.83	7.88	23.24	10.35

SD—standard deviation, MIDAS—Migraine Disability Assessment, HADS—Hospital Anxiety and Depression Scale, OBC—Oral Behaviors Checklist, sig.—*p*-value for the statistical significance of the analyzed differences (see main text for details).

#### Marital status

We did not observe any statistically significant differences in MIDAS (F(1, 1127) = 0.39, *p* = 0.53), HADS-D (F(2,1127) = 0.69, *p* = 0.41), HADS-A (F(2,1127) = 2.81, *p* = 0.09), HADS total (F(2,1127) = 0.29, *p* = 0.59) and OBC (F(2,1126) = 2.55, *p* = 0.07) scores between participants who were in a relationship and those who were single. The details are presented in Table 3.

**Table 3:** Mean scores for the MIDAS, HADS-D, HADS-A, HADS total and OBC in different relationship status groups (SD—standard deviation).

	MIDAS		HADS-D		HADS-A		HADS total		OBC	
	Mean	SD	Mean	SD	Mean	SD	Mean	SD	Mean	SD
In a relationship (n = 820)	21.15	39.00	7.99	4.10	11.91	4.63	19.91	7.95	23.41	10.28
Single (n = 309)	19.39	48.85	8.22	4.21	11.40	4.40	19.62	7.71	22.77	10.54
Sig	*p* = .53		*p* = .41		*p* = .09		*p* = .59		*p* = .07	
Total (n = 1129)	20.67	41.91	8.06	4.13	11.77	4.57	19.83	7.88	23.24	10.35

SD—standard deviation, MIDAS—Migraine Disability Assessment, HADS—Hospital Anxiety and Depression Scale, OBC—Oral Behaviors Checklist, sig.—*p*-value for the statistical significance of the analyzed differences (see main text for details).

#### Education level

Only 7 participants reported having a primary level of education. These respondents were included in the high school group. We observed a statistically significant effect of education level on the HADS-D (F(2,1126) = 5.20, *p* = 0.006), HADS-A (F(2,1126) = 9.06, *p* = 0.0001), HADS total (F(2,1126) = 8.61, *p* = 0.0002) and OBC (F(2,1126) = 8.97, *p* = 0.0001) scores. Participants with higher education levels had lower scores on all of the analyzed measures. While moderate scores were characteristic of people with a college education, the highest scores were identified in people with a high school education. In all cases, the post hoc Tukey test showed that differences between people with higher education and a high school education were statistically significant (all *p* values < 0.005). The differences between people with higher education and a college education and between those with a college education and a high school education were statistically nonsignificant (all *p*'s > 0.09) except for HADS-A. Here, respondents with higher education differed significantly from those with a college education (*p* = 0.048). The difference between the latter group and people with a high school education was statistically nonsignificant (*p* = 0.14). The effect of education was statistically nonsignificant for the MIDAS score (F_Welch_(2, 612.90) = 2.94, *p* = 0.054). The details are presented in Table 4.

**Table 4:** Mean scores for the MIDAS, HADS-D, HADS-A, HADS total and OBC in different education level groups (SD – standard deviation).

	MIDAS		HADS-D		HADS-A		HADS total		OBC	
	Mean	SD	Mean	SD	Mean	SD	Mean	SD	Mean	SD
Higher^I^ (n = 290)	17.52	37.19	7.54	3.86	10.92	4.44	18.46	7.43	21.41	9.85
College^II^ (n = 520)	19.07	36.28	8.01	4.12	11.81	4.53	19.82	7.77	23.21	10.11
High school^III^ (n = 319)	26.12	52.74	8.61	4.34	12.49	4.65	21.09	8.26	24.94	10.90
Sig./pairwise comparisons	n/a		I vs II: p = .35 I vs III: p = .005 II vs III: p = .16		I vs II: p = .048 I vs III: p = .0001 II vs III: p = .14		I vs II: p = .09 I vs III: p = .0002 II vs III: p = .10		I vs II: p = .09 I vs III: p = .0001 II vs III: p = .09	
Total (n = 1129)	20.67	41.91	8.06	4.13	11.77	4.57	19.83	7.88	23.24	10.35

SD—standard deviation, MIDAS—Migraine Disability Assessment, HADS—Hospital Anxiety and Depression Scale, OBC—Oral Behaviors Checklist, sig.—*p* value for the statistical significance of the analyzed differences, n/a—not applicable since the analysis indicated no statistically significant differences (see main text for details).

### Trees for each target variable

#### Assumptions

To determine which groups of people are at a high risk for migraine-related disability, depression, anxiety and oral behaviors, we built separate decision trees for all analyzed variables. We expected each tree to maintain a proper balance between specificity and generalizability.

If a tree is too specific (detailed), the model can be overfitted and may work well with the current dataset but not with the new datasets. An adequate level of generalizability, however, allows us to capture the underlying structure of data and draw conclusions that can be applied to new observations. Considering the above, we decided that the maximum depth of a tree should be 4 and the minimum number of samples to generate leaves should be 10% of the sample size, which was 113 individuals (those numbers were selected arbitrarily).

#### Hospital anxiety and depression scale (HADS) anxiety

**Figure 1:**
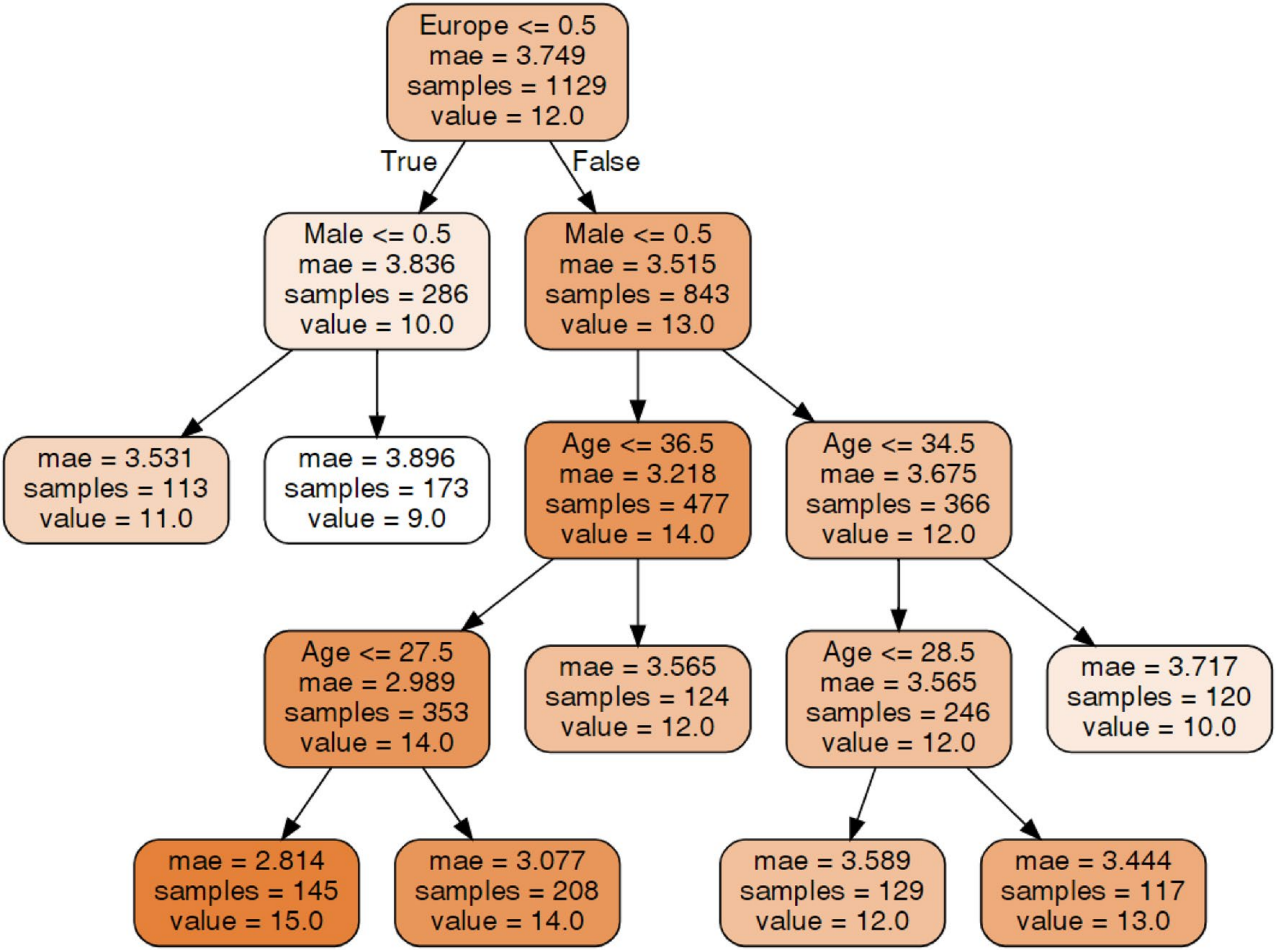
Decision tree for the Hospital Anxiety and Depression Scale (HADS) Anxiety. Source: author-generated tree using the sklearn and graphviz libraries.

An explanation of the decision tree construction and the method of tree interpretation is presented in section "Validation of the classification procedure". With regard to the HADS-A score (Fig. 1), place of residence and gender were important features (higher in Europe, higher and in females). In Europe, age also affected the HADS-A score. Interestingly, older female individuals had lower HADS-A scores (12) than younger female individuals (14), but among males, the highest scores were identified in those between 29 and 34 years old (13). Younger males and older males had relatively lower scores (12 and 10, respectively). The lowest HADS-A score (9.0) was in the group of American males. The highest HADS-A score (15.0) was in European females younger than 27.5 years.

#### Hospital anxiety and depression scale (HADS) depression

**Figure 2:**
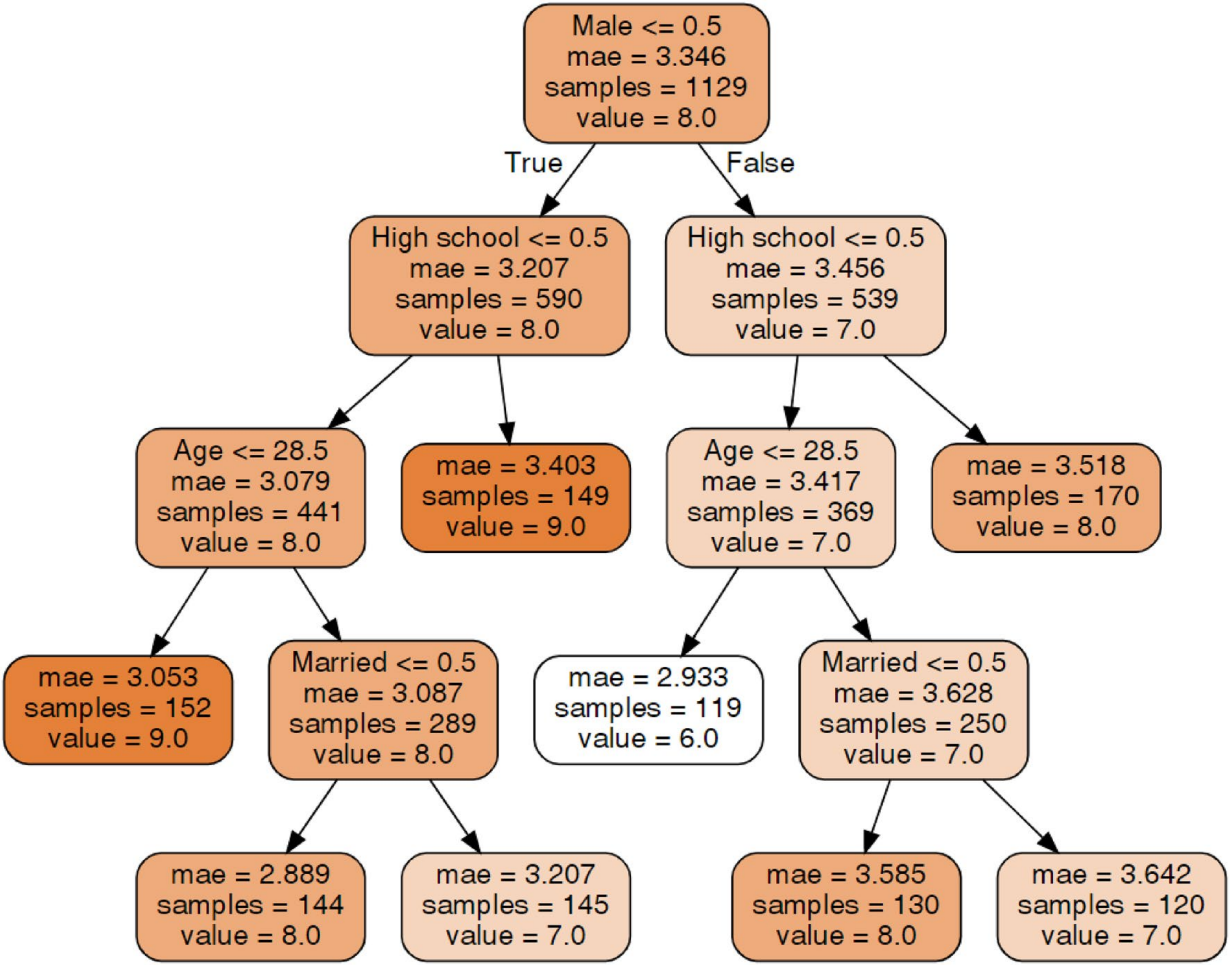
Decision tree for Hospital Anxiety and Depression Scale (HADS) Depression. Source: author-generated tree using the sklearn and graphviz libraries.

An explanation of the decision tree construction and the method of tree interpretation is presented in section "Validation of the classification procedure". In regard to the HADS-D score (Fig. 2), for both males and females, education, age and relationship status were important. Interestingly, among males with higher education levels than high school, younger individuals (< 28.5) had lower HADS-D scores (6.0) than older individuals (≥ 29), who had an average score of 7.0. Among females with the same level of education, younger individuals had higher HADS-D scores (9.0) than older individuals (8.0). Males with higher education levels than high school who were younger than 28.5 years old had the lowest HADS-D scores (6.0). The highest HADS-D scores (9.0) were identified in females with a high school education or those with a relatively higher education level who were younger than 28.5 years.

#### Oral behavior checklist (OBC)

**Figure 3:**
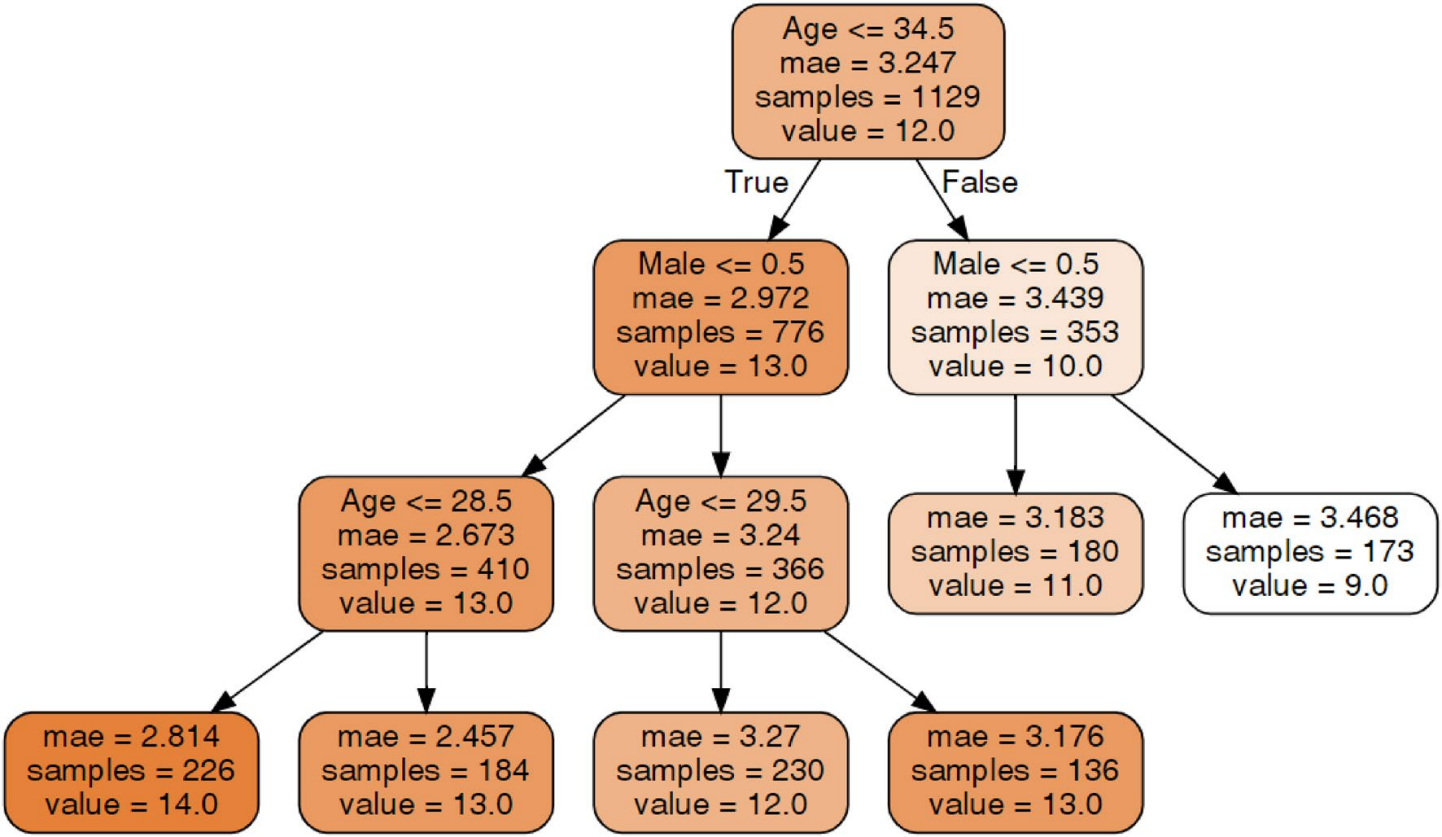
Decision tree for Oral Behavior Checklist (OBC). Source: author-generated tree using the sklearn and graphviz libraries.

An explanation of the decision tree construction and the method of tree interpretation is presented in section "Validation of the classification procedure". In terms of the OBC scores (Fig. 3), age and gender were important. Among females, younger people had higher OBC scores than older people. Among males, middle aged people (30–34 years old) had the highest OBC score (13.0). Younger males (12.0) and older males (9.0) had relatively lower OBC scores, with the latter having the lowest OBC scores in the entire population. The highest OBC score (14.0) was found in younger (under 28.5 years old) females.

#### Migraine disability assessment (MIDAS)

**Figure 4:**
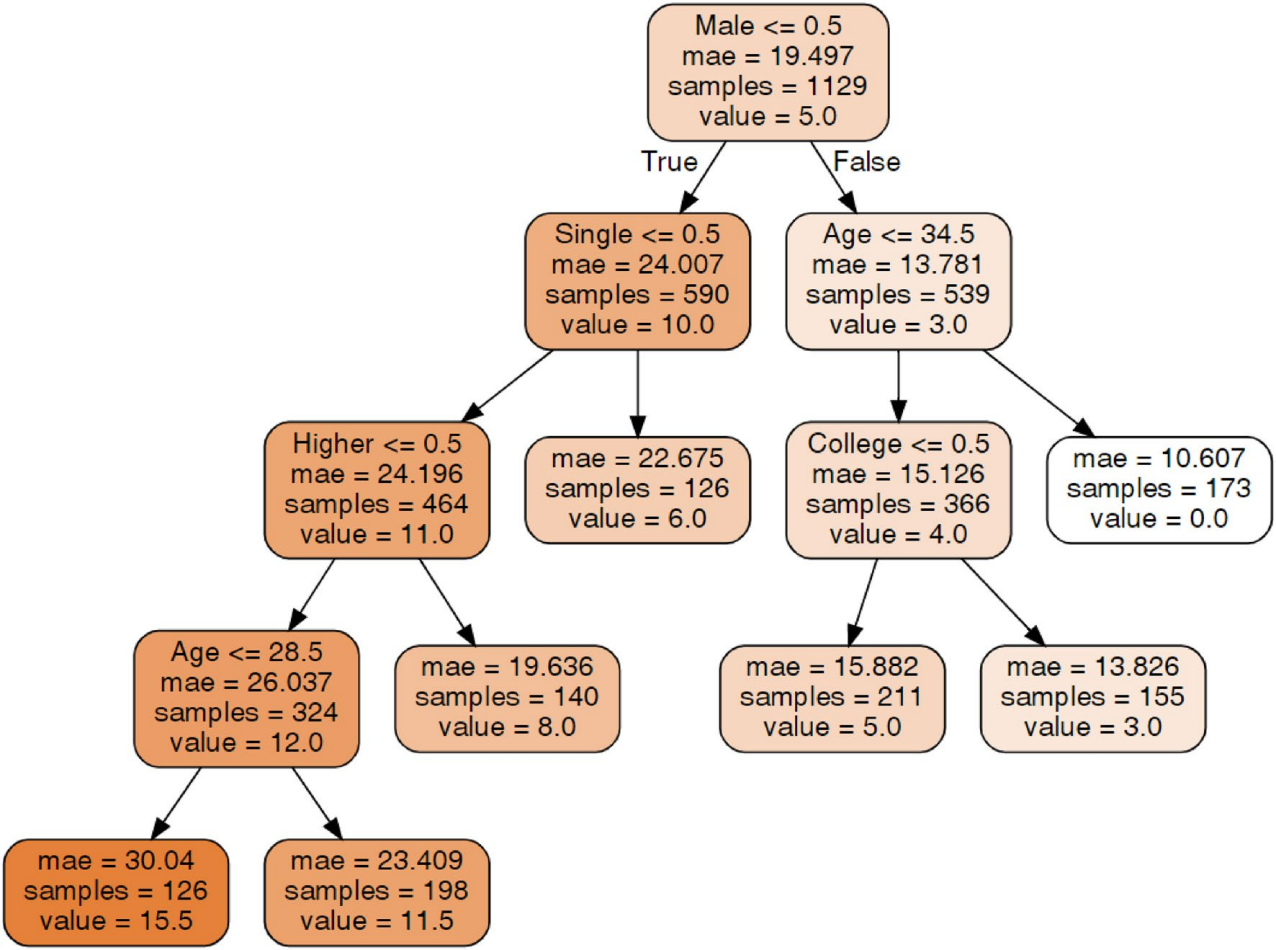
Decision tree for Migraine Disability Assessment (MIDAS). Source: author-generated tree using the sklearn and graphviz libraries.

An explanation of the decision tree construction and the method of tree interpretation is presented in section "Validation of the classification procedure". The tree presented in Fig. 4 shows that among males, age was important for the MIDAS score, but among females, relationship status and education level were important. The tree shows that the lowest MIDAS score, which was 0, was identified in males older than 34.5 years. The highest score (average 15.5) was in females who were not single, had education levels other than higher education and were younger than 28.5 years. The groups with intermediate MIDAS scores can be seen on the tree.

Based on each tree, we calculated the feature importance of each characteristic for each analyzed variable. These calculations are presented in Table 5. The most informative (with highest impact on the analyzed value) features are highlighted.

**Table 5:** Feature importance for each analyzed variable.

	HADS-A	HADS-D	HADS Total	MIDAS	OBC
Age	0.22	0.09	0.18	0.11	**0.71**
College	0.00	0.00	0.00	0.06	0.00
Europe	**0.48**	0.00	0.14	0.00	0.00
High school	0.00	**0.41**	0.00	0.00	0.00
Higher education	0.00	0.00	0.10	0.06	0.00
In a relationship	0.00	0.00	0.00	0.00	0.00
Male	0.30	0.30	**0.57**	**0.64**	0.29
Married	0.00	0.20	0.00	0.00	0.00
Single	0.00	0.00	0.00	0.12	0.00
Total	1.00	1.00	1.00	1.00	1.00

The most informative (with highest impact on the analyzed value) features are indicated in bold.

MIDAS—Migraine Disability Assessment, HADS—Hospital Anxiety and Depression Scale, OBC—Oral Behaviors Checklist.

### Validation of the classification procedure

When the assigning rule was applied to the dataset for Europeans, the most vulnerable group accounted for 27.6% of the population. The most vulnerable group in North America using the same rule accounted for 45.6% of the population. In other words, if the help was provided to the entire population in Europe, 72.4% of the people who received it would not have needed it because they were not in the most vulnerable group. Similarly, in North America, the proportion of people who were not in most susceptible group was 54.5%.

In this step, classifiers were generated separately for Europe and North America. As mentioned in the methods, we used 60% of the data to train the classifiers (create rules). The remaining 40% of the sample was used to test the efficiency of the classifier.

After the classification was applied to the remaining 40% of the sample, we obtained the predicted probability of the respondents belonging to the high-risk group.

As mentioned in the methods, we further selected 1/3 of the individuals from the test sample who were predicted to belong to the group at highest risk. In this subsample, we calculated the precision metric, which is the fraction of the positive respondents (i.e., those who were classified as high-risk individuals), among those selected from the highest risk subsample. The operation was performed separately for North American and European residents. For North American residents, 59.8% of the people were properly classified as the most vulnerable. For Europe, the 44.7% were properly classified.

## Discussion

Although COVID-19 primarily affects physical health, the secondary influence of issues related to the pandemic on mental health should also receive attention. Previously published surveys showed increased symptoms of depression, anxiety, and stress related to COVID-19, as a possible result of psychosocial stressors such as the fear of the disease, the loss of life and economic issues^10–27^. Moreover, the results of the aforementioned studies were heterogeneous, probably because of differences in methods, locations and the timing of the studies with regard to the stage of the pandemic^16^. Only two studies on oral behaviors have been published^28,29^. None of the results of the published surveys assessed headache as a somatic symptom related to the COVID-19 pandemic. However, due to a large amount of worrisome information in social media, the general public is also experiencing overwhelming psychological pressure, which may lead to a variety of psychological conditions, stress-related parafunctional oral behaviors and somatization. Moreover, mental health aspects are relegated to the background due to the heavy burden on local and global health systems imposed by the immediate effects of the pandemic. Therefore, it is essential to identify simple method of identifying the group at high risk of developing psychiatric disorders and deliver early preventive measures or treatment to avoid further consequences. The present online survey was conducted to identify the predictors, risk factors and factors associated with mental disorders (anxiety, depression), headache, and oral parafunctional behaviors among the adult residents of North America and Europe during the COVID-19 pandemic.

As the one of the most important results, in the entire study group, we observed high HADS-A scores. The results are in agreement with data on the prevalence of anxiety during other epidemiological or natural catastrophes, such as the Ebola outbreak^30^, tsunamis^31^ and the September 11th attacks^32^. Moreover, most recent data on mental health problems in China during the COVID-19 outbreak have also shown increased risks of anxiety and depression^19^.

In presented study, we observed higher anxiety scores in younger participants and female. Available literature shows that early-onset generalized anxiety disorder (GAD) is associated with female gender, higher education levels and higher levels of neuroticism, while late-onset GAD is associated with physical illnesses^33^. What is more, gender-based differences in anxiety have been consistently found, and females are approximately twice as likely as males to have mood disorders^34^.

Numerous studies have shown that health and mortality outcomes for married persons are better than those for single persons^35^, especially among men^36^. In a recent study investigating the relationship between marriage and quality of life, single men were found to have a worse quality of life than married men, whereas single women were found to have a better quality of life than married, separate or divorced women^37^. However, data concerning marital status and anxiety are controversial. A higher anxiety level was observed in single individuals than in married persons among patients with epilepsy^38^. However, the level of anxiety was similar in married and single patients receiving palliative radiotherapy^39^. In the present study, we did not observe a relationship between the anxiety score and relationship status.

In presented study, the anxiety score was correlated also to educational level. Patients with higher education levels had lower anxiety scores than those with primary and high school educations. The similar effect of education on anxiety has been also demonstrated previously. The Hunt study showed that a higher educational level may protect against anxiety and depression^40^. Also, Cekirdekci and Bugan showed higher anxiety scores in patients with lower education levels in the population diagnosed with cardiac syndrome X^41^.

Also, the place of residence seems to influence the anxiety score. In the available literature, the prevalence of anxiety has been shown to be higher in North America than in European countries^42^. It is worth noting that we also observed the effect of place of residence on anxiety. In the present study, North American respondents had generally higher anxiety levels than respondents from Europe. On the other hand, taking into account the decision trees analysis and predictors, the highest anxiety level was identified in European females who were younger than 27.5 years.

In presented study, we tried also to establish the similar relations for depression scores, as mood disorders are highly prevalent in the global population, with prevalence ranging from 5.4 to 7.8%^43^. Available literature shows that the lifetime prevalence of depression in females is twice that in males^44^. Females with depression tend to have a younger age of onset^45^, longer duration^46^ more severe and recurrent episodes^47^ and lower quality of life^48^ than male patients. Education level has been associated with the risk of depression^49^. However, this relationship is not consistent, and some studies have shown that a lower education level is not related to a higher prevalence of major depression^50^.

In the present study, effects of gender and education level on the depression score were observed. The highest depression score was observed in females with a high school education or with an education level other than high school who were younger than 28.5 years. Taking into account the place of residence, depression scores were similar in North American and European respondents.

Another aspect studied in this manuscript was headache. As the headache can be the associated with somatization and is highly prevalent worldwide^51^. Numerous studies in the general population have consistently demonstrated that headache is more prevalent in women than in men^52,53^. The most important risk factors for headache include the overuse of acute migraine medication, ineffective acute treatment, obesity, depression, stressful life events, age, and low education level^53^. In the present study, females and subjects with lower education levels had higher MIDAS scores. As the effect of gender and education level on migraine has been previously described^51,52^, the results of this study are in line with the findings of previous studies. Taking into account decision trees analysis, the highest MIDAS scores were observed in the group of non-single females with education levels other than higher education who were younger than 28.5 years.

Another studied in the presented study aspects were potentially stress-related oral behaviors. To the best of our knowledge, this is the first study to investigate the prevalence of oral behaviors during the COVID-19 pandemic. Oral behaviors are frequently observed in the general population and can lead to serious clinical implications including temporomandibular disorders and orofacial pain^54^. The relationship between oral behaviors and temporomandibular disorder has been reported by several authors in children, adolescents and adults^54–57^. Oral parafunctions include teeth clenching, lip biting, thumb sucking, nail biting and other oral habits. Bruxism is the most common oral motor activity and is anticipated to be present in 31% of the general population^58^. Nail biting and holding objects in the mouth are other oral parafunctions observed frequently in children and adolescents^59^. Winocur et al.^55^ found that biting hard objects and nail biting were associated with tired jaws in adolescent females. Atsü demonstrated that TMD signs and symptoms were relatively more frequent in the adolescent female group (47.8%), and these results may be explained by biological differences, hormone levels and higher pain sensitivity in women^59^. In the present study, the effects of gender, age, place of residence, and education level on the OBC score were observed. Similar to the results regarding anxiety levels, younger females with lower education levels were in the highest risk group for parafunctional oral behavior.

The highest OBC scores were observed in females younger than 28.5 years.

The design of the study was thorough and enabled the authors to obtain results online in an easy way and in a short time period. It allowed us to define risk groups rapidly, which, in the future, may allow the establishment of precisely targeted risk groups and the provision of the necessary prophylactic or treatment measures to the highest-risk population, thereby preventing the development of mental disturbances during this and other global crises. It is very important to achieve scientific advances even when access to patients is difficult, and assistance cannot be provided to everyone. The present study is the first to consider potentially stress-related parafunctional oral behaviors, the occurrence of headaches and the prevalence of mental disorders during the COVID-19 pandemic. Moreover, this is the first study to highlight the risk factors for mental disorders and high-risk groups who could potentially develop mental disorders during global crises. The strength of the study is the fact that it was conducted on a large and representative group of respondents and compared residents in two different continents: North America and Europe. The questionnaires used in the study were validated, established and highly specific tools. The obtained results are novel, interesting and clinically useful.

Despite its novelty and many strengths, this study is not without some limitations. First, the study was performed as an online survey, which, despite widespread access to the Internet, could possibly have defined or limited the study group. It is expected that this form of data obtaining would be more available for younger respondents as the elderly may be less technology proficient. This could influence reliability of the data including potential bias. Additionally, the fact that the survey was conducted in English was a limitation, especially for residents of Europe.

The present study demonstrated showed levels of anxiety, depression, headache, and oral behaviors during the COVID-19 pandemic in both North America and European residents. For the first time, we have also shown increased levels of oral parafunctional habits during the COVID-19 pandemic, which may result in an increased prevalence of orofacial pain and temporomandibular disorders in the future. Therefore, health care systems should be prepared for more patients with mental disorders, headache, orofacial pain and temporomandibular disorders during the current pandemic and future global crises. The results obtained in this study facilitated the identification of the group at highest risk for the mentioned secondary effects of the pandemic. This group was composed of females younger than 28.5 years old, especially those who were single, less well educated and living in Europe. These results indicate the need to perform further research in this population. Determining this risk group may allow the implementation of screening tests and the faster implementation of preventive and treatment measures, with the aim of reducing the long-term negative effects of this and future global crises. Due to the fact that in the times of almost every crisis, performing screening tests and access to large populations could be very difficult, in authors’ opinion, the clinical recommendations from the presented study findings would be performing screening for the occurrence of psycho-emotional disturbances and somatization first in the defined highest risk groups. This will allow faster detection of people presenting disturbing symptoms and faster and more accurate implementation of interventions.

## Methods

This study was conducted in accordance with the principles of the Declaration of Helsinki. The study was approved by the Ethics Committee of Wroclaw Medical University in Poland (ID: KB-302/2020). All the study participants provided informed consent before being included in the study.

### Data collection procedure

To collect the data, the authors created an online questionnaire on the Google Original Platform—Google Forms because (1) it is the platform with which they have the greatest experience and (2) according to them, it is the most user-friendly for both researchers and respondents. The questionnaire was posted on Reddit, an American social news aggregation platform that also allows users to be involved in discussions. The authors posted links to the questionnaire on several Reddit pages called “subreddits”, including local American and European forums as well as SARS-CoV-2-related forums. Reaching out to these internet communities and explaining why such data are important enabled rapid data collection—in 3 days, from March 22th to March 25th, 2020, the authors collected 1642 answers. It is worth mentioning that the Redditors (as Reddit users call themselves) who took part in the survey were satisfied with their participation, and many of them decided to share the link to the questionnaire with their families or friends. The authors chose to post the questionnaire on Reddit because they noticed that subreddits were thriving in the first quarter of 2020; for example, according to subredditstats.com, a website with statistical data about subreddits, /r/Coronavirus, which is currently the largest SARS-CoV-2-related subreddit, was created on 20th of January 2020. It had 6 subscribers that day, but by the end of April 2020, it had grown to have more than two million subscribers. The questionnaire was anonymous, and the authors did not collect any data that allowed them to identify the respondents.

### Questionnaires

#### Author sociodemographic survey

The sociodemographic portion of the survey asked basic questions about gender , age, place of residence (name of country), marital status (in a relationship, married or single), education level (primary school, high school or college or higher), and existing medical conditions. As the educational level information could vary between specific countries due to differences between the independent educational systems, we tried to mention all the possible universal types/levels of education: primary school, high school, college graduate, higher education (professional or post-graduate level). Then, for the purposes of statistical analysis, on the basis of presented possible answers, we created 3 types of categories: primary education (primary school), secondary education (high school or college graduate) and higher education (professional or post-graduate level). This allowed for taking into account the problem of differences in individual educational systems of individual countries.

#### Hospital anxiety and depression scale (HADS)

The HADS is a widely used self-assessment of anxiety and depressive symptoms, focusing mostly on the cognitive and psychological aspects^60–62^. Somatic concerns and physical symptoms are not assessed by this scale. It is commonly used in general medical populations as well as in healthy populations^63^. The psychometric properties, including the internal consistency, discriminatory ability, validity and test–retest correlations, are considered satisfactory; thus, the HADS is one of the most commonly used self-assessment questionnaires for anxiety/depression symptom screening^62^.

The HADS consists of a total of 14 items in 2 separate subscales: anxiety (HADS-A) and depression (HADS-D), each of which includes 7 items. All items were scored by the participant using a Likert scale (4 points, from 0 to 3 points). The total score varies from 0 to 42 points, and both subscale scores vary from 0 to 21 points.

The originally recommended cutoff scores for the subscales were as follows: a score from 0 to 7 indicates a noncase, a score from 8 to 10 indicates a possible case, and a score from 11 to 21 indicates a probable case^63^. Currently, the categorization system includes more groups: 0 –7, normal; 8 –10, mild; 11–15, moderate; and > 16, severe^61^.

In this study, scores of 11 or more were considered to indicate a “high risk of anxiety/depression”, according to the cutoff values described above.

#### Migraine disability assessment (MIDAS)

The MIDAS is a short, 5-item tool designed for the rapid assessment of the consequences of migraine for a patient, focusing on time lost (in terms of lack of productivity) due to the headache. The patient indicates the number of days with significant disability due to migraines during the last 3 months before the assessment. The score is obtained by summing days mentioned in the responses to the 5 items, and this total score is classified in one of four clinical groups: little or no disability (0–5 days), mild disability (6–10 days), moderate disability (11–20 days) and severe disability (21 days or more)^64,65^.

All properties, including the internal consistency, test–retest correlations and validity, are considered satisfactory and have been confirmed in several studies^65,66^.

In this study, scores of 21 or more were considered to indicate a “high risk of headache” with a significant impact of those headaches on daily functioning.

#### Oral behavior checklist (OBC)

The OBC is a self-assessment tool designed for the evaluation of the frequency of different oral behaviors during the day or at night. It consists of 21 items, out of which 2 refer to night-time behaviors, while the rest refer to daily oral function. For each item, a participant provides an answer describing the frequency of this behavior: during the night (how many nights in a week such behavior appears) or during the day (none of the time/a little of the time/some of the time/most of the time/all of the time). For each item, a score of 0–4 points is assigned, yielding a total sum in the range from 0 to 84 points. The score is interpreted as follows: 0—no risk of parafunctional oral activity, 1–24—low risk of parafunctional oral activity, 25–84—high risk of parafunctional oral activity.

During the design of the study, the internal consistency, test–retest correlations and validity were found to be good, and the OBC is the tool most commonly used for the assessment of oral behaviors^67^.

In this study, a score of 25 points or more was used as a cutoff value for a high risk of parafunctional oral activity.

### Target group definitions

Given that the HADS does not include questions on somatic concerns, we defined several target “high-risk” groups by combining a score indicating a high risk of mental health issues (HADS-D/A) with a score indicating a high risk of somatic/physical issues (the MIDAS or OBC). In this way, 4 different high-risk target groups were established:

- anxiety and headaches (HADS-A score of 11 or more AND MIDAS score of 21 or more);
- anxiety and oral parafunctional activity (HADS-A score of 11 or more AND OBC score of 25 or more);
- depression and headaches (HADS-D score of 11 or more AND MIDAS score of 21 or more);
- depression and oral parafunctional activity (HADS-D score of 11 or more AND OBC score of 25 or more).

Based on the definitions of these high-risk target groups, in the third stage of the research, we defined the group at highest risk of negative effects and applied a classification algorithm to predict if the likelihood of belonging to such a group was associated with any basic characteristics (age, gender, place of residence, relationship status and education level).

In this way, the possible combinations of the risks of two different mental disorders with the risks of two different somatic/physical manifestations were exhaustively analyzed. It is noteworthy that the use of the HADS does not allow any diagnosis of depressive or anxiety disorders; it only indicates a high risk of anxiety/depressive symptoms^62^ and suggests that professional/institutional help should be administered.

### Statistical analysis

We analyzed our data in three stages. First, we analyzed the background characteristics of the entire sample. When our data did not satisfy the assumptions of standard parametric analyses, we used either nonparametric tests or relevant alternatives to classic parametric methods. Accordingly, Spearman's rank correlation coefficients were used to assess the relationships between nonnormally distributed continuous variables (participant age and MIDAS, HADS, OBC scores). To test for significant differences between groups of respondents defined by categorical variables (i.e., respondent gender, place of residence, relationship status, education level) we used both classic one-way ANOVA (if variances in the compared groups were homoscedastic) or Welch's one-way ANOVA (if group variances were heteroscedastic). We report the results from Welch's ANOVA analyses with the appropriate remarks. If Welch's ANOVA indicated statistically significant results and more than two groups were analyzed, post hoc pairwise comparisons were performed with the Games-Howell test. Post hoc pairwise comparisons for the classic one-way ANOVA were conducted with the Tukey test.

### Decision tree-based analysis

In the second stage, we generated a regression decision tree to identify the multidimensional dependencies among all characteristics identified in the first stage (age, gender, place of residence, relationship status and education level) and analyzed the values (MIDAS, HADS and OBC).

The primary goal of using decision trees is to create a model that predicts the value of a target variable by learning simple decision rules inferred from the data features. A decision tree is one of many predictive modeling methods. The clear advantage of the decision tree model over other methods (linear regression, supported vector machines, artificial neural networks and others) is its graphical representation enabling the straightforward interpretation of the rules explaining dependencies among variables. Unlike in the standard procedure, in which one subset of data is used to train the model and the second subset is used to validate its efficiency, here we built a model on the entire available dataset to visualize and discern the influence of the variables on the analyzed values. In this study, we used the common Python (version 3.7.4) programming language and generated decision trees with the CART algorithm. The authors used decision tree implementation provided in the scikit-learn library (one of the most popular machine learning libraries on GitHub. https://scikit-learn.org/).

Decision trees can be built only with continuous (numerical) variables that require prior transformation, and the categorical features (gender, place of residence, relationship status and education level) were encoded as a numeric array. We used the label binarization method to transform each categorical variable (LabelBinarizer method in sklearn.preprocessing, https://scikit-learn.org/stable/modules/generated/sklearn.preprocessing.LabelBinarizer.html). If the variable had 2 possible values, e.g., gender (female, male) one result feature is generated. In the example of gender, the result would be 1 if person is a male and 0 if the person is a female. When the initial variable has more options, e.g., education level (higher, college, high school), the result of label binarization is a set of features for each possible option, and they are assigned the value of 1 if the original row has option and 0 when it has a different one.

**Figure 5:**
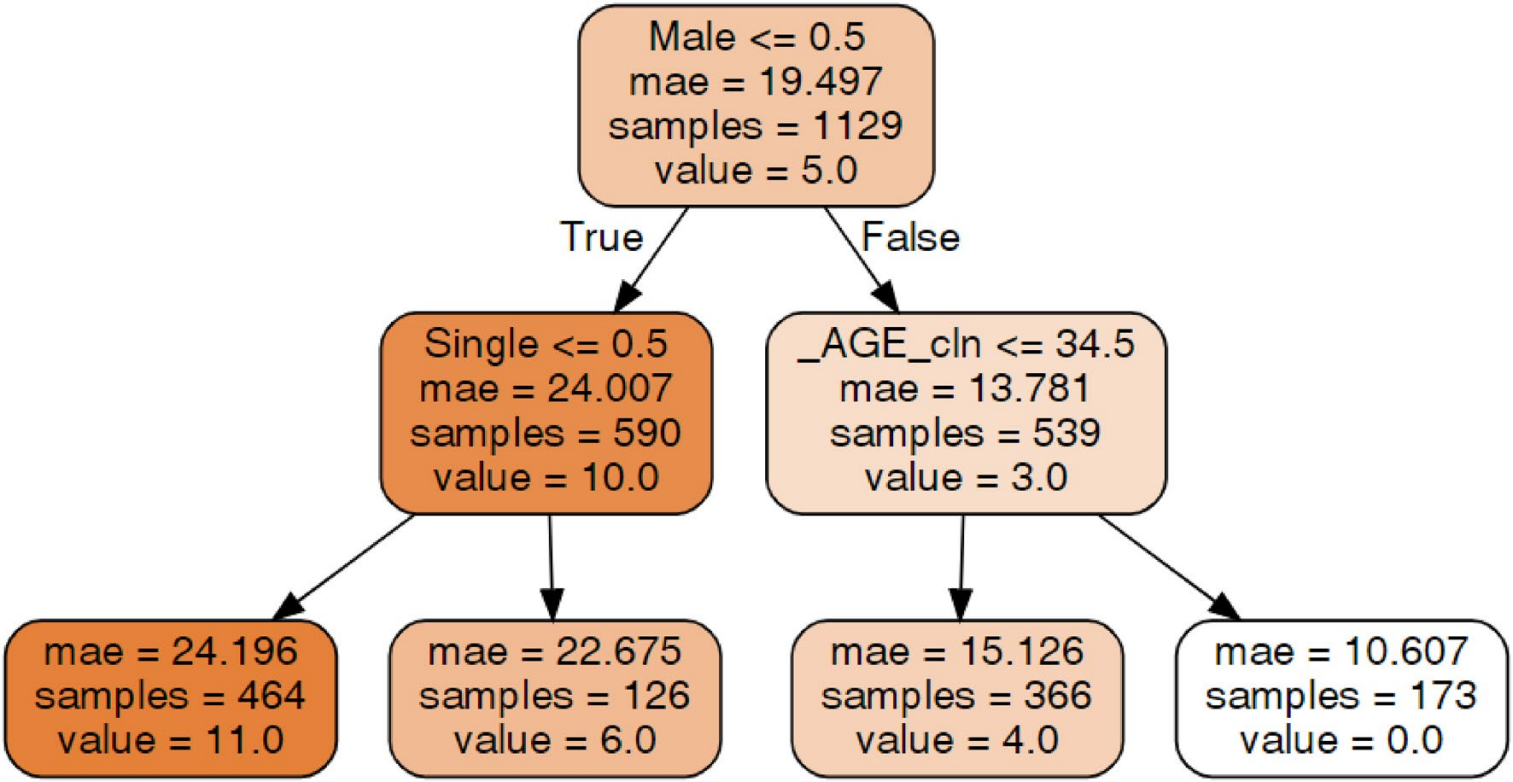
Example of a decision tree with the Migraine Disability Assessment (MIDAS) target variable. Source: author-generated tree using the sklearn and graphviz libraries.

Although decision trees are a very informative and compelling method of data exploration and data mining^68^, they are not very common in the biomedical literature; therefore, in Fig. 5, a hypothetical decision tree is presented, and brief guidelines for the appropriate interpretation of the results obtained from the trees generated in our study are presented.

Each decision tree includes root nodes, subnodes and leaf nodes. They are connected with branches. Nodes include the predicates (e.g., male ≤ 0.5), the size of a sample (samples), the predicted value on a current level (value) and the mean absolute error (mae). Prediction with classification trees is performed by navigating down the tree through the logical results of the predicates until a leaf is reached^69^. If the logical test of the predicate results in “true”, we follow the left-hand option; otherwise, we should follow the right-hand option. The predicted value is presented in a leaf node. If we would like to use the presented tree to predict the MIDAS score for a single (single = 1) woman (male = 0), we need to answer Male ≤ 0.5 (the predicate in the root node). In our case, the logical value “true” is returned. Then, we follow the “true” branch (left-hand side). The predicted value for females is 10.0. The next step in the prediction process is to answer single (≤ 0.5). In the given example (single = 1), the result is “false”, so we follow the right-hand side. The final prediction is 6.0. We also know that there are 126 observations in the initial dataset of single females. The indicated mae is high, so this prediction is likely inaccurate.

The mae is a measure of the error between observations expressing the same phenomenon. It is calculated with the following formula:

$$MAE = \mathop \sum \limits_{i = n}^{n} \frac{{\left| {y_{i} - x_{i} } \right|}}{n}$$

where, y_i_—prediction, x_i_—actual value, n—sample size.

Having generated a decision tree, we are able to evaluate the importance of each feature in the prediction process. Feature importance evaluation always pertains to a generated decision tree. To perform such an evaluation, the Gini importance score is calculated. Splits in a decision tree are determined by choosing the feature and splitting criterion that result in the greatest reduction in total impurity, which ultimately indicates the importance of that feature in the specific tree. A split that generates a large decrease in impurity is considered important; therefore, variables used to determine important splits are also considered important. Based on this idea, the importance for each variable X in terms of the reduction in impurity is computed as the sum of all the measures of the decrease in impurity at all nodes in the tree at which a split occurs based on X^70^.

When the Gini importance score is 1, it means that one feature is sufficient to predict the analyzed value. If it is 0, such a feature is not represented in a tree at all. The sum of all Gini scores for all features is 1. The higher the Gini score, the more informative (important) a feature is (the more influence it has on an analyzed value).

### Validation of the prediction of the high-risk group

The third stage of the analysis was the validation of how accurate the prediction of the high-risk was and involved the application of the knowledge acquired in previous stages. The target group definition presented earlier was used in this stage.

The primary aim of identifying dependencies among all characteristics selected in the first stage (age, gender, place of residence, marital status and education level) and the analyzed scales was to determine the most vulnerable individuals to enable the precise and efficient targeting of the provision of support. In this stage of the analysis, we validated the efficiency of the developed classification procedure. This was accomplished in several steps. First, we defined a rule assigning a person to the most vulnerable group according to the mentioned cutoff points for each of the analyzed measures. Consequently, the rule assigning a given person to the most vulnerable group was as follows:

- If
- (HADS ANXIETY > 10 AND MIDAS > 20) OR.
- (HADS ANXIETY > 10 AND OBC > 24) OR.
- (HADS DEPRESSION > 10 AND MIDAS > 20) OR.
- (HADS DEPRESSION > 10 AND OBC > 24).
- THEN
- EXPOSED = True.
- ELSE
- EXPOSED = False.

This rule was based on the 4 different high-risk target groups defined earlier.

Having identified this group, we assumed that relevant interventions should be delivered separately to the entire populations of North America and Europe. However, the efficiency of such an approach is questionable since a great deal of effort will probably be devoted to diagnosing and providing help to people who do not actually need it. The efficiency of such an approach is calculated as the percent of the population that truly needs the help.

The next step involved building a classifier using part of the initial dataset (the so-called training set) to train the model. The training set was 60% of the initial dataset. During the training process, we fed the algorithm the basic characteristics of the respondents: gender, relationship status, and education level. The result was whether an individual belonged to the most vulnerable group.

The goal of the classifier is to assign a person who has not been diagnosed to a specific group (i.e., i) most vulnerable; ii) the rest of population), knowing only the mentioned basic characteristics and lacking an actual diagnosis.

After the classifier is trained, its efficiency should be verified. Classification quality is calculated based on the remaining part of the initial dataset that was not used in the training process. The test set was composed of the remaining 40% of respondents. During testing, classification was performed on that 40% of the samples. The classifier predicts the probability that respondents belong to the high-risk group. Assuming that help is delivered primarily to the group predicted by the classifier as the most vulnerable persons, we calculated the efficiency and compared it to the initial situation where we assumed the delivery of interventions to the entire population. We assumed that support should be provided to 1/3 of the population due to resource-related limitations, which is why the results will be calculated for the group of respondents for whom the classification predicted the highest probability of a high level of risk.

The classifiers were created separately for North America and Europe and were validated in the corresponding sets.

## Data Availability

The data that support the findings of this study are available from the corresponding author upon reasonable request.

## References

[1] Yang X (2020). Clinical course and outcomes of critically ill patients with SARS-CoV-2 pneumonia in Wuhan, China: a single-centered, retrospective, observational study. Lancet Respir. Med..

[2] Zhou P (2020). A pneumonia outbreak associated with a new coronavirus of probable bat origin. Nature.

[3] Huang C (2020). Clinical features of patients infected with 2019 novel coronavirus in Wuhan, China. Lancet.

[4] Wu F (2020). A new coronavirus associated with human respiratory disease in China. Nature.

[5] Li Q (2020). Early transmission dynamics in Wuhan, China, of novel coronavirus–infected pneumonia. N. Engl. J. Med..

[6] World Health Organization. WHO Director-General's opening remarks at the media briefing on COVID-19–11 March 2020. https://www.who.int/director-general/speeches/detail/who-director-general-s-opening-remarks-at-the-media-briefing-on-covid-19-11-march-2020 (2020).

[7] Borges do Nascimento, I. J. et al. Novel coronavirus infection (COVID-19) in humans: a scoping review and meta-analysis. *J. Clin. Med.* **9,** 941 (2020). 10.3390/jcm9040941 PMC7230636 32235486

[8] CDC. Are you at higher risk for severe illness? https://www.cdc.gov/coronavirus/2019ncov/specificgroups/highriskcomplications.html (2020).

[9] Li W (2020). Progression of mental health services during the COVID-19 outbreak in China. Int. J. Biol. Sci..

[10] Wang C (2020). Immediate psychological responses and associated factors during the initial stage of the 2019 Coronavirus Disease (COVID-19) epidemic among the general population in China. Int. J. Environ. Res. Public Health.

[11] Liu N (2020). Prevalence and predictors of PTSS during COVID-19 outbreak in China hardest-hit areas: gender differences matter. Psychiatry Res..

[12] Qiu J (2020). A nationwide survey of psychological distress among Chinese people in the COVID-19 epidemic: implications and policy recommendations. Gen. Psychiatry.

[13] Zhou SJ (2020). Prevalence and socio-demographic correlates of psychological health problems in Chinese adolescents during the outbreak of COVID-19. Eur Child Adolesc Psychiatry.

[14] Tee ML (2020). Psychological impact of COVID-19 pandemic in the Philippines. J Affect Disord.

[15] Magnavita N, Chirico F (2020). Headaches, personal protective equipment, and psychosocial factors associated with COVID-19 pandemic. Headache.

[16] Holmes EA (2020). Multidisciplinary research priorities for the COVID-19 pandemic: a call for action for mental health science. Lancet Psychiatry.

[17] Li J (2020). Anxiety and depression among general population in China at the peak of the COVID-19 epidemic. World Psychiatry.

[18] Cao W (2020). The psychological impact of the COVID-19 epidemic on college students in China. Psychiatry Res..

[19] Gao J (2020). Mental health problems and social media exposure during COVID-19 outbreak. PLoS ONE.

[20] Lei L (2020). Comparison of prevalence and associated factors of anxiety and depression among people affected by versus people unaffected by quarantine during the COVID-19 epidemic in Southwestern China. Med. Sci. Monit..

[21] Tang W (2020). Prevalence and correlates of PTSD and depressive symptoms one month after the outbreak of the COVID-19 epidemic in a sample of home-quarantined Chinese university students. J. Affect. Disord..

[22] Xiao H, Zhang Y, Kong D, Li S, Yang N (2020). Social capital and sleep quality in individuals who self-isolated for 14 days during the coronavirus disease 2019 (COVID-19) outbreak in January 2020 in China. Med. Sci. Monit..

[23] Yuan S (2020). Comparison of the indicators of psychological stress in the population of hubei province and non-endemic provinces in china during two weeks during the coronavirus disease 2019 (COVID-19) outbreak in February 2020. Med. Sci. Monit..

[24] Fullana MA, Hidalgo-Mazzei D, Vieta E, Radua J (2020). Coping behaviors associated with decreased anxiety and depressive symptoms during the COVID-19 pandemic and lockdown. J. Affect. Disord..

[25] González-Sanguino C (2020). Mental health consequences during the initial stage of the 2020 Coronavirus pandemic (COVID-19) in Spain. Brain Behav. Immun..

[26] Zhang Y, Ma ZF (2020). Impact of the COVID-19 pandemic on mental health and quality of life among local residents in Liaoning Province, China: a cross-sectional study. Int. J. Environ. Res. Public Health.

[27] Hamel, L. et al. KFF coronavirus poll: March 2020. https://www.kff.org/coronavirus-covid-19/poll-finding/kff-coronavirus-poll-march-2020/ (2020).

[28] Almeida-Leite CM, Stuginski-Barbosa J, Conti PCR (2020). How psychosocial and economic impacts of COVID-19 pandemic can interfere on bruxism and temporomandibular disorders?. J. Appl. Oral Sci..

[29] Emodi-Perlman A (2020). Temporomandibular disorders and bruxism outbreak as a possible factor of orofacial pain worsening during the COVID-19 pandemic-concomitant research in two countries. J. Clin. Med..

[30] Shultz JM, Baingana F, Neria Y (2015). The 2014 Ebola outbreak and mental health: current status and recommended response. JAMA.

[31] van Griensven F (2006). Mental health problems among adults in tsunami-affected areas in Southern Thailand. JAMA.

[32] Updegraff JA, Silver RC, Holman EA (2008). Searching for and finding meaning in collective trauma: results from a national longitudinal study of the 9/11 terrorist attacks. J. Pers. Soc. Psychol..

[33] Rhebergen D (2017). Admixture analysis of age of onset in generalized anxiety disorder. J. Anxiety Disord..

[34] Weisberg RB (2009). Overview of generalized anxiety disorder: epidemiology, presentation, and course. J. Clin. Psychiatry.

[35] Hu Y, Goldman N (1990). Mortality differentials by marital status: an international comparison. Demography.

[36] Ben-Shlomo Y, Smith GD, Shipley M, Marmot MG (1993). Magnitude and causes of mortality differences between married and unmarried men. J. Epidemiol. Community Health.

[37] Han KT, Park EC, Kim JH, Kim SJ, Park S (2014). Is marital status associated with quality of life?. Health Qual. Life Outcomes.

[38] Wang F-L (2017). Influence of marital status on the quality of life of Chinese adult patients with epilepsy. Chin. Med. J..

[39] Nieder C, Kämpe TA (2018). Does marital status influence levels of anxiety and depression before palliative radiotherapy?. Vivo.

[40] Bjelland I (2008). Does a higher educational level protect against anxiety and depression? The HUNT study. Soc. Sci. Med..

[41] Cekirdekci EI, Bugan B (2019). Level of anxiety and depression in cardiac syndrome X. Med. Princ. Pract..

[42] Kessler RC (2007). The global burden of anxiety and mood disorders: putting the European Study of the Epidemiology of Mental Disorders (ESEMeD) findings into perspective. J. Clin. Psychiatry.

[43] Steel Z (2014). The global prevalence of common mental disorders: a systematic review and meta-analysis 1980–2013. Int. J. Epidemiol..

[44] Oldehinkel AJ, Bouma EM (2011). Sensitivity to the depressogenic effect of stress and HPA-axis reactivity in adolescence: a review of gender differences. Neurosci. Biobehav. Rev..

[45] Schuch JJ, Roest AM, Nolen WA, Penninx BW, de Jonge P (2014). Gender differences in major depressive disorder: results from the Netherlands study of depression and anxiety. J. Affect. Disord..

[46] Marcus SM (2005). Gender differences in depression: findings from the STAR*D study. J. Affect. Disord..

[47] Brailean A, Curtis J, Davis K, Dregan A, Hotopf M (2020). Characteristics, comorbidities, and correlates of atypical depression: evidence from the UK Biobank Mental Health Survey. Psychol. Med..

[48] Kornstein SG (2000). Gender differences in chronic major and double depression. J. Affect. Disord..

[49] Saraceno B, Levav I, Kohn R (2005). The public mental health significance of research on socio-economic factors in schizophrenia and major depression. World Psychiatry.

[50] Molarius A, Granström F (2018). Educational differences in psychological distress? Results from a population-based sample of men and women in Sweden in 2012. BMJ Open.

[51] Stovner LJ, Andree C (2010). Prevalence of headache in Europe: a review for the Eurolight project. J. Headache Pain.

[52] Baykan B (2007). The burden of headache in neurology outpatient clinics in Turkey. Pain Pract..

[53] Lipton RB, Bigal ME (2005). Migraine: epidemiology, impact, and risk factors for progression. Headache.

[54] Gavish A, Halachmi M, Winocur E, Gazit E (2000). Oral habits and their association with signs and symptoms of temporomandibular disorders in adolescent girls. J. Oral Rehabil..

[55] Winocur E, Gavish A, Finkelshtein T, Halachmi M, Gazit E (2001). Oral habits among adolescent girls and their association with symptoms of temporomandibular disorders. J. Oral Rehabil..

[56] Wieckiewicz M (2014). Prevalence and correlation between TMD based on RDC/TMD diagnoses, oral parafunctions and psychoemotional stress in Polish university students. Biomed. Res. Int..

[57] Lobbezoo F (2018). International consensus on the assessment of bruxism: Report of a work in progress. J. Oral Rehabil..

[58] Alamoudi N (2001). Correlation between oral parafunction and temporomandibular disorders and emotional status among saudi children. J. Clin. Pediatr. Dent..

[59] Atsü SS, Güner S, Palulu N, Bulut AC, Kürkçüoğlu I (2019). Oral parafunctions, personality traits, anxiety and their association with signs and symptoms of temporomandibular disorders in the adolescents. Afr Health Sci.

[60] Zigmond AS, Snaith RP (1983). The hospital anxiety and depression scale. Acta Psychiatr. Scand..

[61] Beekman E, Verhagen A (2018). Clinimetrics: hospital anxiety and depression scale. J. Physiother..

[62] Smarr KL, Keefer AL (2011). Measures of depression and depressive symptoms: Beck Depression Inventory-II (BDI-II), Center for Epidemiologic Studies Depression Scale (CES-D), Geriatric Depression Scale (GDS), Hospital Anxiety and Depression Scale (HADS), and Patient Health Questionnaire-9 (PHQ-9). Arthritis Care Res..

[63] Snaith, R. & Zigmond, A. S. *The Hospital Anxiety and Depression Scale Manual* (Nfer-Nelson, Windsor, 1994).

[64] Stewart WF, Lipton RB, Dowson AJ, Sawyer J (2001). Development and testing of the Migraine Disability Assessment (MIDAS) questionnaire to assess headache-related disability. Neurology.

[65] Stewart WF (1999). An international study to assess reliability of the Migraine Disability Assessment (MIDAS) score. Neurology.

[66] Edmeads J, Láinez JM, Brandes JL, Schoenen J, Freitag F (2001). Potential of the Migraine Disability Assessment (MIDAS) questionnaire as a public health initiative and in clinical practice. Neurology.

[67] Kaplan SE, Ohrbach R (2016). Self-report of waking-state oral parafunctional behaviors in the natural environment. J. Oral Facial Pain Headache.

[68] Sheng, B. & Gengxin, S. Data Mining in census data with CART. *2010 3rd International Conference on Advanced Computer Theory and Engineering(ICACTE)*, 3, V3–260-V3–264 (2010).

[69] Liu L, Ozsu MT (2009). Encyclopedia of Database Systems.

[70] Nembrini S, König IR, Wright MN (2018). The revival of the Gini importance?. Bioinformatics.

