# Author Correction: Identification of risk groups for mental disorders, headache and oral behaviors in adults during the COVID-19 pandemic

**DOI:** 10.1038/s41598-021-97151-4

**Published:** 2021-08-27

**Authors:** Mieszko Wieckiewicz, Dariusz Danel, Maciej Pondel, Joanna Smardz, Helena Martynowicz, Tomasz Wieczorek, Grzegorz Mazur, Robert Pudlo, Gniewko Wieckiewicz

**Affiliations:** 1 Department of Experimental Dentistry, Wroclaw Medical University, 26 Krakowska St., 50‑425 Wroclaw, Poland; 2 Department of Anthropology, Hirszfeld Institute of Immunology and Experimental Therapy, Polish Academy of Sciences, 12 Rudolfa Weigla St., 53‑114 Wroclaw, Poland; 3 Department of Business Intelligence in Management, Wroclaw University of Economics and Business, 118‑120 Komandorska St., 53‑345 Wroclaw, Poland; 4 Department of Internal Medicine, Occupational Diseases, Hypertension and Clinical Oncology, Wroclaw Medical University, 213 Borowska St., 50‑556 Wroclaw, Poland; 5 Department and Clinic of Psychiatry, Wroclaw Medical University, 10 Pasteura St., 50‑367 Wroclaw, Poland; 6 Chair and Clinical Department of Psychiatry in Tarnowskie Gory, Medical University of Silesia, 49 Pyskowicka St., 42‑612 Tarnowskie Gory, Poland

Correction to: *Scientific Reports* 10.1038/s41598-021-90566-z, published online 26 May 2021

The original version of this Article contained an error in Table 5.

The value given for the category “Male” for the “HADS-A” variable, “0.30”, was incorrectly given as “0.00.”

The incorrect and correct values are given below.

Incorrect:

**Table:** 

	HADS-A	HADS-D	HADS Total	MIDAS	OBC
Male	0.00	0.30	**0.57**	**0.64**	0.29

Correct:

**Table:** 

	HADS-A	HADS-D	HADS Total	MIDAS	OBC
Male	0.30	0.30	**0.57**	**0.64**	0.29

The original Article has been corrected.

